# Collision Tumor of the Appendix

**DOI:** 10.7759/cureus.38638

**Published:** 2023-05-06

**Authors:** Álvaro Morillo Cox, Tatiana Fernandez Trokhimtchouk, Luis F Flores, Diego Viteri, Olmedo Mancero, Estefanie S Otanez

**Affiliations:** 1 General Surgery, Universidad Internacional del Ecuador/Axxis Hospital, Quito, ECU; 2 General Surgery, Axxis Hospital, Quito, ECU; 3 Pathology, Axxis Hospital, Quito, ECU

**Keywords:** collision tumour, appendicitis, appendiceal tumor, neuroendocrine tumor, mucinous neoplasm, acute abdomen

## Abstract

This report discusses the case of a 20-year-old female patient who presented with acute abdominal pain, nausea, and vomiting. Initial laboratory analyses suggested an inflammatory process, but imaging studies failed to reveal pathologies. The patient underwent a diagnostic laparoscopy, which showed a thickened and multicystic appendix with signs of acute inflammation. Pathology indicated a positive cytology for malignancy, with a grade 1 mixed well-differentiated neuroendocrine tumor (NET) and high-grade mucinous neoplasm identified in the middle and distal thirds of the appendix. Finding both tumors in the same patient is extremely rare and has been reported in a few cases. The case emphasizes the importance of considering appendiceal tumors in the differential diagnosis of acute abdominal pain, even in young patients, and highlights the value of laparoscopy in their diagnosis. The early detection and appropriate management of appendiceal tumors are crucial for improving patient outcomes.

## Introduction

Primary appendiceal tumors are rare, with a reported incidence of approximately 1.2 cases per 100,000 per year in the United States [1]. They comprise several histologic types, which are broadly characterized as epithelial or neuroendocrine neoplasms. Appendiceal mucinous neoplasms (AMNs) are of epithelial lineage and account for less than 1% of all gastrointestinal cancers. They have a female predominance and are usually diagnosed in the sixth decade of life [2]. Neuroendocrine tumors (NETs), which are less common according to some studies, also have a higher incidence in female patients, but during their 40s [3]. Both these cancers can be aggressive, particularly if they are poorly differentiated [4,5]. 

The majority of appendiceal NETs, as well as AMNs, are found incidentally at the time of appendectomy (1-2% of all specimens), or during radiologic or endoscopic evaluation for unrelated complaints. As most NETs are submucosal and located in the distal third of the appendix, they are unlikely to cause obstruction. Patients with AMNs are often asymptomatic or may present with nonspecific symptoms, including acute or chronic right lower quadrant abdominal pain, abdominal palpable mass, or gastrointestinal bleeding [6]. 

Collision (proliferation of two different cell lines) or combined (multidirectional differentiation of a single tumor) neoplasms can rarely occur. As these are exceptional cases, no consensus on their approach and management exists [7]. 

Here, we present the case of a 20-year-old woman who presented to the emergency room with abdominal pain suggesting appendicitis. Imaging studies did not show clear evidence of an inflammatory appendicular process or any other underlying pathologies. The patient underwent a diagnostic laparoscopy, which revealed a thickened and multicystic appendix with signs of acute inflammation. Appendectomy was performed uneventfully and histopathology analysis revealed a 1.2 cm grade 1 well-differentiated NET and a 0.6 cm high-grade mucinous neoplasm.

This report highlights the importance of considering appendiceal tumors in patients with suspected acute appendicitis. Also, it opens a discussion on how to proceed based on intraoperative and postoperative histopathologic findings.

## Case presentation

A 20-year-old female, with a history of polycystic ovarian syndrome treated with combined oral contraceptives, presented to the emergency room with 36 hours of moderate abdominal pain. She described it as initially diffuse but later exacerbated and localized to the right lower quadrant with accompanying nausea and three episodes of vomiting.

The patient had normal vital signs, and no fever was recorded. Physical examination revealed tenderness upon light palpation on the right iliac fossa, and McBurney’s and Blumberg’s signs were positive.

Laboratory analyses revealed leukocytosis, neutrophilia, and elevated CRP. Hemoglobin and hematocrit levels were within the normal range. An appendicitis inflammatory response score of 6 points was calculated. Relevant results and normal ranges are listed in Table 1.

**Table 1:** Laboratory Analyses

Parameter	Value	Normal range
Leukocytes	11820	4320 - 10540 / mm3
Neutrophils	77.5	50 - 70 %
Hemoglobin	14.4	12.7 - 162 g / dL
Hematocrit	42.5	38 - 47 %
C-reactive protein (CRP)	42	<10 md / dL
Lipase	24	13 - 69 U / L
Amylase	54	28 - 102 U / L

**Figure 1:**
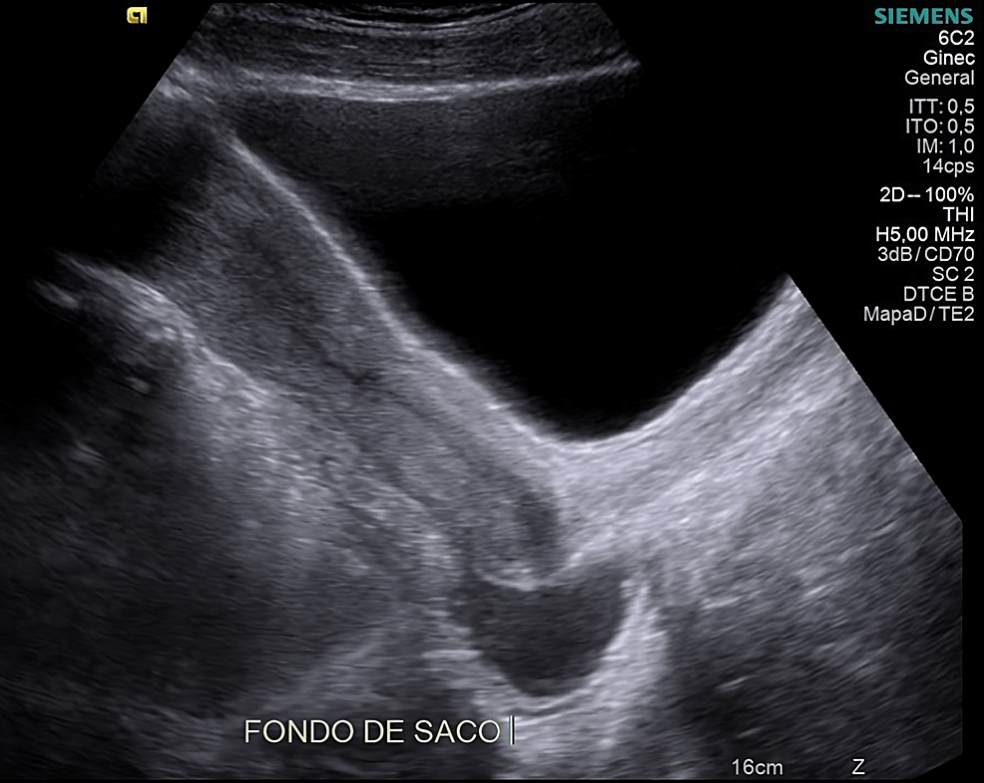
Pelvic ultrasound showing laminar fluid in Douglas’ pouch

A pelvic ultrasound was performed, which showed minimal amounts of free fluid in the pelvic region (Figure 1). No signs of adnexal or uterine pathology were observed, and there were no findings compatible with acute appendicitis.

**Figure 2:**
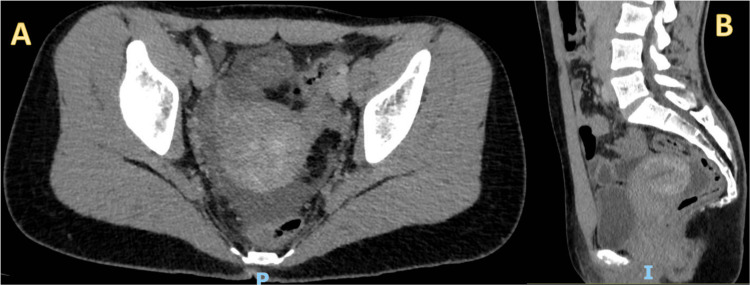
A: Axial view of contrast abdominopelvic CT showing homogeneous free fluid in the rectouterine pouch, extending to the right iliac fossa. B shows a sagittal view.

An abdominal CT was ordered, which could not clearly identify the vermiform appendix but revealed homogeneous free fluid in the rectouterine pouch, extending to the right iliac fossa (Figure 2) suggesting an inflammatory pelvic process with no clear primary cause.

An acute abdomen was diagnosed, following which the patient was taken to the operating room for a diagnostic laparoscopy. The appendix was located in a pelvic position and its middle and distal thirds appeared thickened with a multicystic appearance. The base looked normal. Approximately 50 mL of inflammatory free fluid was found in the Douglas pouch, while other structures appeared macroscopically normal. Appendectomy was performed without complications, and no liquid from the cysts was spilled in the peritoneal cavity. The patient had an uneventful postoperative recovery and was discharged home on the first day after the procedure. 

**Figure 3:**
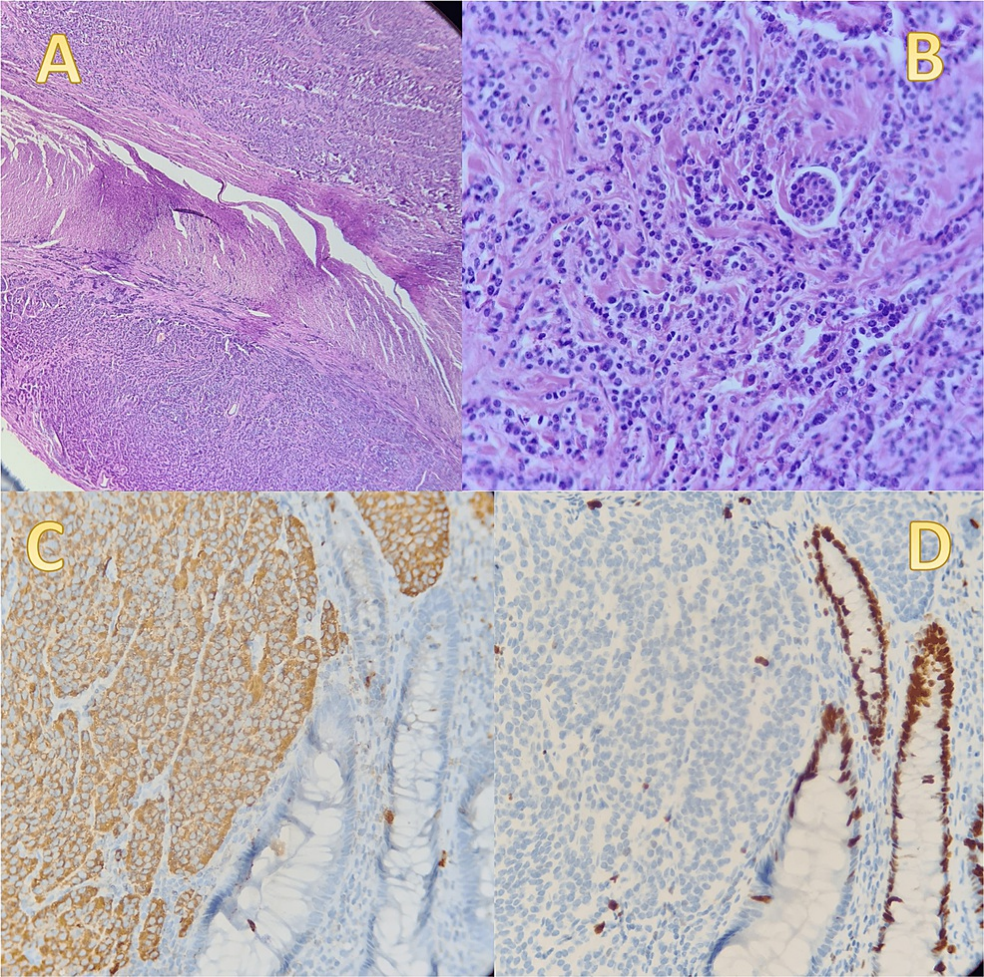
A 1.2 cm well-differentiated neuroendocrine tumor (A) shows low power magnification. (B) shows high power magnification: bland and uniform nuclei. Immunohistochemistry: (C) shows chromogranin A positive in 2/3 of tumor cells, synaptophysin (not shown) was positive in 1/3 of tumor cells. (D) Ki67 less than 1%, consistent with a grade 1 tumor

**Figure 4:**
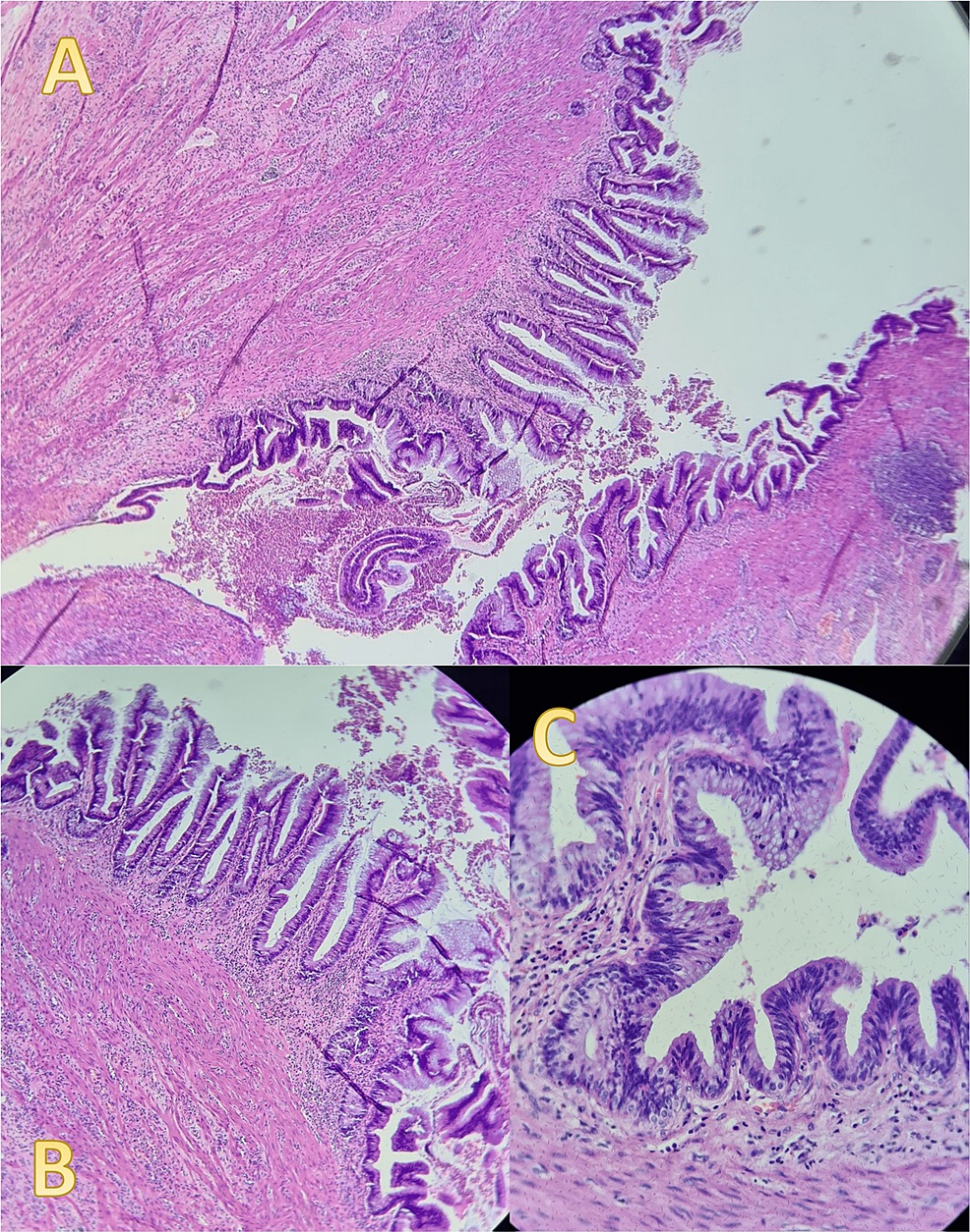
A 0.6 cm high-grade appendiceal mucinous neoplasm. (A) shows low power magnification with no infiltration. (B) and (C) high power magnification showing high-grade dysplasia, convoluted architectural patterns, micropapillarity, cribriforming, and acute transmural infiltration

According to the pathology report, a collision of a grade 1 well-differentiated neuroendocrine tumor (NET) (Figure 3) and a high-grade appendiceal mucinous neoplasm (HAMN) were identified (Figure 4), both located within the middle and distal thirds of the appendix. The neuroendocrine component was located in the subserosa, while the HAMN was found in the lamina propria. The report stated that resection margins were free. The appendix also showed signs of acute gangrenous inflammation. The staging was reported as pT3 for the NET and pTis for the HAMN. No perineural, lymphovascular, or mesoappendicular invasion was detected, and since no liquid from the cysts spilled in the peritoneal cavity, staging did not change.

## Discussion

Appendicular neoplasms are infrequent, comprising 1% of colorectal cancers, and the most common types are NET and AMN. They may present as an incidental finding in imaging studies, during surgery, or in the pathology report, or they can be diagnosed after an episode of acute appendicitis [8]. 

Collision and combined appendiceal tumors are even more uncommon [9]. A collision tumor is defined as two or more different neoplasms occurring synchronously in the same organ with a clear transition zone, as a result of the proliferation of two cellular lines. There are less than 30 cases of concomitant AMN and NET of the appendix reported in the literature, and most of them show the presence of these two most frequent neoplasias [10]. 

HAMN is the term that describes a true neoplasm, confined to the muscularis propria, with a high degree of epithelial dysplasia present. It produces abundant mucin and exhibits expansile growth which may cause loss of muscular wall and would explain the development of symptoms. They lack infiltrative invasion due to which their management is closer to that of low-grade AMNs [11].

Patients with HAMN confined to the appendix, which has not ruptured, and is completely resected by appendectomy do not require a completion surgery. The prognosis after appropriate treatment is excellent, with an overall five-year survival rate of 86%. Such is the case of the patient we report [8].

On the other hand, appendiceal NETs are usually detected during a histological examination after an appendectomy, as they do not typically present with symptoms. Current evidence suggests that well-differentiated grade 1 NETs between 1-2 cm, in the absence of mesoappendicular invasion, positive margins, or angioinvasion, can be treated with appendectomy alone. With this approach, the overall five-year survival rate reaches up to 100% [12].

As there are no guidelines or consensus for the management of collision appendicular tumors due to the rarity of the presentation, the approach should be individualized. In most of the reported cases, treatment is based on the recommendations for the individual neoplasms, and decisions are made based on the one with a poorer prognosis, as well as frequency and duration of follow-up [10]. In this patient, considering that the histopathology report showed a collision pattern, appendectomy alone seems to be a safe alternative without increasing morbimortality and healthcare costs and does not require follow-up [13].

## Conclusions

In conclusion, this case report highlights the rare occurrence of collision appendiceal tumors and the importance to know how to proceed with these patients. Early detection and appropriate management of appendicular neoplasia are critical for improving outcomes and reducing the risk of metastasis. 

Diagnostic laparoscopy remains a crucial element in the armamentarium of the general surgeon when facing acute abdominal pain, even when imaging studies fail to show the etiology.

Surgical resection of appendicular tumors remains the primary treatment option, although the extent of surgery depends on the tumor location, size, and histological type. Chemotherapy and/or radiation therapy may also be used in certain cases, such as those with aggressive or metastatic tumors.
